# Preoperative globulin‐to‐albumin ratio predicts outcome after curative resection in patients with gastric cancer

**DOI:** 10.1002/ags3.12200

**Published:** 2018-08-31

**Authors:** Takayuki Shimizu, Mitsuru Ishizuka, Norisuke Shibuya, Genki Tanaka, Akihito Abe, Taku Aoki, Keiichi Kubota

**Affiliations:** ^1^ Second Department of Surgery Dokkyo Medical University Tochigi Japan

**Keywords:** curative resection, gastric cancer, globulin‐to‐albumin ratio, inflammation‐based prognostic system, postoperative survival

## Abstract

**Aim:**

The globulin‐to‐albumin ratio (GAR) is useful for prognostication of patients with various cancers. However, the significance of GAR in gastric cancer (GC) remains unclear. Our purpose was to investigate the relationship between the GAR and outcome after curative resection in GC patients.

**Methods:**

Three‐hundred and seventy‐six patients who had undergone curative resection for GC were retrospectively reviewed. Univariate and multivariate analyses using the Cox proportional hazard model were performed to detect clinical characteristics that correlated with overall survival (OS), and their cut‐off values were identified using receiver operating characteristic (ROC) curve analyses. Kaplan–Meier analysis and log‐rank test were used for comparison of OS and relapse‐free survival (RFS).

**Results:**

Multivariate analysis using 17 clinical characteristics selected by univariate analyses revealed that GAR (>0.80/≤0.80) was significantly associated with OS (hazard ratio [HR], 2.305; 95% CI, 1.122‐4.735; *P *=* *0.023), as well as lymph node metastasis (presence/absence) (HR, 2.417; 95% CI, 1.077‐5.426; *P *=* *0.032), neutrophil‐to‐lymphocyte ratio (>2.7/≤2.7) (HR, 2.368; 95% CI, 1.138‐4.930; *P *=* *0.002), and serosal invasion (presence/absence) (HR, 3.443; 95% CI, 1.048‐11.31; *P *=* *0.042). Kaplan–Meier analysis and log‐rank test demonstrated that the OS and RFS of patients with a high GAR (>0.80) were significantly poorer than those with low GAR (≤0.80).

**Conclusions:**

The GAR is a useful predictor of postoperative outcome among GC patients undergoing curative resection.

## 1 INTRODUCTION

Several studies have shown that the combination of globulin and albumin can predict the outcome of patients with various cancers such as nasopharyngeal cancer, non‐small‐cell lung cancer, and upper tract urothelial carcinoma.^1^, ^2^, ^3^ The globulin‐to‐albumin ratio (GAR) is the ratio of the serum globulin level and the serum albumin level, both of which are routinely measured at preoperative term. Like the Glasgow Prognostic Score (GPS) and Neutrophil‐to‐lymphocyte ratio (NLR), the GAR reflects the systemic inflammatory response as interleukin‐6 (IL‐6) induces differentiation of B‐lymphocytes into plasma cells, which produce immunoglobulins and reduce the level of serum albumin.^4^, ^5^ Therefore, the GAR should predict the outcome after curative resection for gastric cancer (GC), as has been shown for other cancers.^1^, ^2^, ^3^

In fact, a recent study demonstrated that the combination of globulin and albumin was able to predict outcome in GC patients undergoing curative surgery.^6^ Although the previous study mentioned that the combination of globulin and albumin stratified disease‐free survival (DFS) of GC patients with lymph node invasion or serosal invasion, and this combination was a good indication for postoperative adjuvant chemotherapy in such GC patients,^6^ the relationship between the GAR and tumor‐node‐metastasis (TNM) stage was unclear in that study. Therefore, in order to prove the significance of GAR in prognostication of GC patients, the relationship between the GAR and each TNM stage of GC patients should be investigated.

Furthermore, it was unclear whether there was an association between GAR and relapse‐free survival (RFS), as only overall surgery (OS) and DFS.^6^ However, OS and DFS after surgery are unable to indicate whether the GAR is significantly associated with tumor progression including recurrence after surgery, because the GAR would also reflect the nutritional status of GC patients. In order to resolve these two issues, it is necessary to investigate whether the GAR is associated with not only TNM stage but also RFS after surgery.

In the present study, therefore, we investigated not only the relationship between GAR and TNM stage but also the relationship between GAR and both OS and RFS after curative surgery in GC patients using a single‐institution clinical database.

## 2 MATERIALS AND METHODS

We retrospectively reviewed 376 patients with newly diagnosed GC who had undergone curative resection at the Second Department of Surgery, Dokkyo Medical University Hospital, between January 2003 and December 2015. We excluded patients with unresectable GC or patients who had undergone combined resection of other organs. Because the occurrence of anastomotic leakage has been reportedly associated with outcome after total gastrectomy for GC,^7^ we additionally excluded patients with postoperative anastomotic leakage. None of the patients exhibited clinical evidence of infection or other inflammatory conditions, and none had received preoperative chemotherapy or irradiation. All procedures were performed by a single well‐trained surgical team.

Routine laboratory measurements including the serum level of albumin and globulin were carried out on the day of admission. We used these preoperative data to determine the GAR in this study.

### 2.1 Definition of inflammation‐based prognostic systems

We calculated each patient's GPS as follows: Patient with elevated level of CRP (>1.0 mg/dL) and hypoalbuminemia (<3.5 g/dL) was assigned a score of 2, patient who exhibited one of these abnormalities was assigned a score of 1, and patient with no abnormalities was assigned a score of 0.^8^ The NLR was calculated as the patient's neutrophil level (% or number of neutrophils) divided by the lymphocyte level (% or number of lymphocytes).^9^ The GAR was calculated as: [the serum total protein level (g/dL)‐the serum albumin level (g/dL)] divided by the serum albumin level (g/dL). The serum total protein level included both the serum globulin level and the serum albumin level.

### 2.2 Definition of operative curability

On the basis of the General Rules for Japanese classification of gastric carcinoma (Japanese Gastric Cancer Association, 3rd English Edition), residual tumors are diagnosed as: R0, no residual tumor; R1, microscopic residual tumor (positive resection margin or cancer cells evident on peritoneal cytology as CY1); R2, macroscopic residual tumor.^10^ On the basis of this definition, curative resection is defined as R0.

### 2.3 Definition of prognostic nutritional index

The prognostic nutritional index (PNI) was calculated as 10 × serum albumin level (g/dL) + 0.005 × total lymphocyte count (/mm^3^).^11^

### 2.4 Definition of TNM stage

We used the TNM classification of Malignant Tumours Eighth Edition edited by the Union for International Cancer Control (UICC) for determining the TNM stage.^12^

### 2.5 Definition of tumor location

On the basis of the General Rules for Japanese classification of gastric carcinoma, the stomach is divided anatomically into three portions—upper (U), middle (M), and lower (L)—by lines connecting points on the lesser and greater curvatures.^10^ If more than one part is involved, all involved portions should be recorded in descending order of degree of involvement, the part containing the bulk of the tumor being listed first, for example, UM, UML, or ML.^10^ Tumor extension into the esophagus or duodenum is recorded as “others” in each case.

### 2.6 Postoperative adjuvant chemotherapy for advanced gastric cancer

On the basis of the Japanese gastric cancer treatment guidelines 2014, patients with stage II and III GC, except for pT1 and pT3N0, are eligible for postoperative adjuvant chemotherapy for prevention of recurrence after surgery.^13^, ^14^ Oral administration of tegafur‐gimestat‐otastat potassium (S‐1) is standard practice for postoperative adjuvant chemotherapy.^13^, ^14^ On the basis of the Japanese gastric cancer treatment guidelines 2014, administration of S‐1 after curative resection has been performed at our institution.^13^, ^14^

### 2.7 Statistical analysis

Data are presented as medians and interquartile ranges. Intergroup differences were analyzed using the chi‐squared test or the Mann–Whitney *U*‐test, as appropriate. Hazard ratio (HR) with 95% confidence intervals (CIs) was calculated by univariate and multivariate analyses using the Cox proportional hazards model. To identify clinical characteristics that were closely related to OS, multivariate analysis was performed using clinical characteristics shown to have a *P*‐value of <0.05 in the univariate analysis. Kaplan–Meier analysis and log‐rank test were used to compare the OS and RFS of the groups. All statistical analyses were performed using the SPSS software package (version 23.0; IBM Co., New York, NY, USA), and differences with a *P*‐value of <0.05 were considered statistically significant.

**Figure 1:**
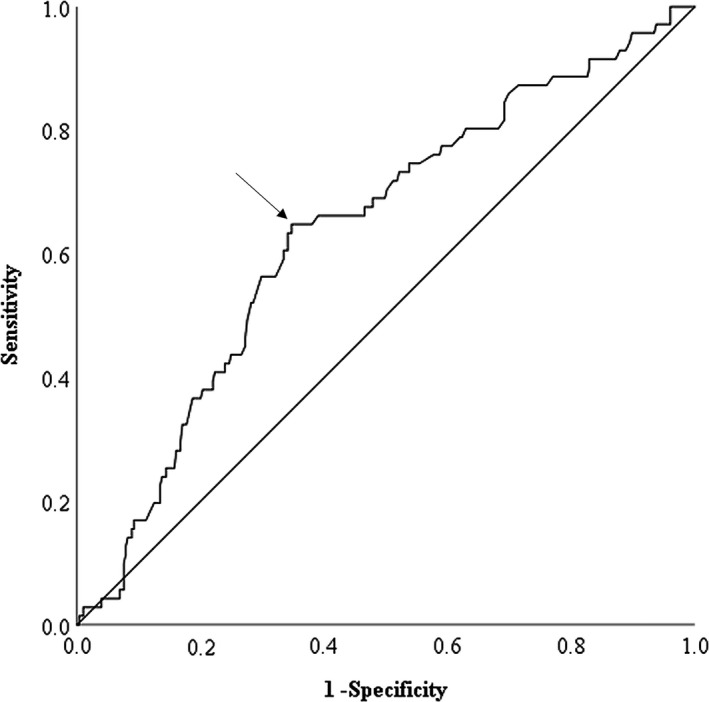
The receiver operating characteristic (ROC) curve shows the optimal cut‐off value of globulin‐to‐albumin ratio (GAR) for patients with gastric cancer. The arrow shows the most prominent point of the ROC curve. The area under the ROC curve of GAR for OS is 0.676

The cut‐off values for the various clinical characteristics were determined using receiver operating characteristic (ROC) curve analyses. The recommended cut‐off values for the characteristics were defined using the most prominent point on the ROC curve (Youden index = maximum [sensitivity‐(1‐specificity)]),^15^ and we also calculated the area under the ROC (AUROC) curve. The optimal cut‐off value for GAR was the 0.80, which provided a sensitivity of 64.8%, a specificity of 62.6%, and an AUROC curve of 0.637 (Figure 1). All other cut‐off values were defined using ROC curve analyses, including as age (year), body mass index (BMI) (kg/m^2^), maximum tumor size (cm), NLR, platelet count (×10^4^/mm^3^), the serum levels of carbohydrate antigen 19‐9 (CA19‐9) (U/mL), carcinoembryonic antigen (CEA) (ng/mL) and globulin (g/dL), PNI and white blood cell (WBC) count (×10^3^/mm^3^), except for the serum levels of albumin (g/dL) and CRP (mg/dL). The AUROC curve for each characteristic was also calculated.

## 3 RESULTS

We enrolled 376 patients (269 male, 107 female), including 216 patients with a low GAR (≤0.80) and 160 with a high GAR (>0.80). Table 1 shows the clinical characteristics of the patients in the two GAR groups. The chi‐squared test revealed significant intergroup differences in GPS (0/1/2), liver cirrhosis (absence/presence), lymphatic invasion (absence/presence), serosal invasion (absence/presence), TNM stage (II, III/I), and venous invasion (absence/presence).

**Table 1:** Relationships between clinical characteristics and GAR in patients with gastric cancer

Variable	GAR ≤ 0.8 (*n* = 216) (57.4%)	GAR > 0.8 (*n* = 160) (42.6%)	*P*‐value
Gender			
Female	68 (18.1%)	39 (10.4%)	0.131
Male	148 (39.4%)	121 (32.2%)	
GPS			
0	200 (53.2%)	79 (21.0%)	<**0.001**
1	16 (4.3%)	61 (16.2%)	
2	0 (0.0%)	20 (5.3%)	
Location			
U	23 (6.1%)	15 (4.1%)	0.089
UM	68 (18.1%)	55 (14.6%)	
M	35 (9.3%)	10 (2.7%)	
ML	8 (2.1%)	8 (2.1%)	
L	74 (19.7%)	64 (17.0%)	
Others	8 (2.1%)	8 (2.1%)	
Liver cirrhosis			
Absence	215 (57.2%)	150 (39.9%)	**0.001**
Presence	1 (0.2%)	10 (2.7%)	
Lymphatic invasion			
Absence	100 (26.6%)	54 (14.4%)	**0.017**
Presence	115 (30.6%)	104 (27.7%)	
Not available	1 (0.2%)	2 (0.5%)	
Lymph node metastasis			
Absence	161 (42.8%)	107 (28.5%)	0.104
Presence	55 (14.6%)	53 (14.1%)	
Number of tumor			
1	195 (51.9%)	135 (36.0%)	0.084
≥2	21 (5.5%)	25 (6.6%)	
Operation			
Distal gastrectomy	133 (35.4%)	82 (21.8%)	0.078
Total gastrectomy	83 (22.0%)	77 (20.6%)	
Proximal gastrectomy	0 (0.0%)	1 (0.2%)	
Pathological differentiation			
Well or moderately	66 (22.1%)	65 (21.7%)	0.092
Others	101 (33.8%)	67 (22.4%)	
Postoperative adjuvant chemotherapy			
Absence	127 (33.8%)	103 (27.4%)	0.686
Presence	40 (10.6%)	29 (7.7%)	
Not available	49 (13.0%)	28 (7.5%)	
Postoperative complication			
CD grade 0	165 (43.9%)	102 (27.2%)	0.091
CD grade I	5 (1.3%)	10 (2.7%)	
CD grade II	28 (7.5%)	25 (6.6%)	
CD grade III	14 (3.7%)	17 (4.5%)	
CD grade IV	2 (0.5%)	4 (1.1%)	
CD grade V	2 (0.5%)	2 (0.5%)	
Serosal invasion			
Absence	164 (43.6%)	94 (25.0%)	<**0.001**
Presence	52 (13.8%)	66 (17.6%)	
TNM Stage			
I	152 (40.4%)	86 (22.9%)	**0.004**
II	33 (8.8%)	36 (9.6%)	
III	31 (8.2%)	38 (10.1%)	
Venous invasion			
Absence	126 (33.5%)	64 (17.1%)	**0.001**
Presence	89 (23.7%)	94 (25.0%)	
Not available	1 (0.2%)	2 (0.5%)	

Chi‐squared test. The bold of *P*‐value means statistical significant value.

CD, Clavian‐Dindo; GAR, globulin‐to‐albumin ratio; GPS, Glasgow prognostic score; TNM, Tumor‐node‐metastasis.

Table 2 shows the clinico‐laboratory characteristics for the two GAR groups. The Mann–Whitney *U*‐test revealed significant intergroup differences in age; maximum tumor size (cm); PNI; the serum levels of albumin (g/dL), CA19‐9 (U/mL), CEA (ng/mL), CRP (mg/dL), and globulin (g/dL); survival period (day); and white blood cell count (×10^3^/mm^3^).

**Table 2:** Relationships between clinico‐laboratory characteristics and GAR in patients with gastric cancer

Variable	GAR ≤ 0.8 (*n* = 216) (57.4%)	GAR > 0.8 (*n* = 160) (42.6%)	*P*‐value
Age (years)	65 (58‐72)	71 (65‐77)	<**0.001**
Albumin (g/dL)	4.0 (3.8‐4.3)	3.5 (3.1‐3.8)	<**0.001**
BMI (kg/m^2^)	22.6 (20.7‐24.9)	22.6 (20.2‐24.8)	0.619
CA19‐9 (U/mL)	7 (4‐13)	10 (5‐25)	**0.011**
CEA (ng/mL)	2.1 (1.4‐3.5)	2.5 (1.7‐4.0)	**0.031**
CRP (mg/dL)	0.10 (0.10‐0.30)	0.30 (0.10‐0.60)	<**0.001**
Globulin (g/dL)	2.7 (2.5‐2.9)	3.3 (2.9‐3.6)	<**0.001**
Maximum tumor size (cm)	3.5 (2.5‐5.1)	4.5 (3.0‐6.5)	**0.006**
NLR	2.0 (1.5‐2.9)	2.2 (1.6‐2.9)	0.497
Operation bleeding (mL)	284 (158‐490)	313 (172‐576)	0.286
Platelet count (×10^4^/mm^3^)	21.9 (18.1‐25.8)	22.8 (18.5‐29.2)	0.153
Prognostic nutritional index	49.1 (46.0‐51.8)	43.6 (38.8‐47.4)	<**0.001**
Survival period (day)	1582 (641‐2605)	1044 (375‐2036)	**0.014**
WBC count (×10^3^/mm^3^)	5.8 (4.8‐6.7)	6.1 (4.7‐7.6)	**0.011**

Median (IQR), Mann–Whitney *U*‐test. The bold of *P*‐value means statistical significant value.

BMI, body mass index; CA19‐9, carbohydrate antigen 19‐9; CEA, carcinoembryonic antigen; CRP, C‐reactive protein; GAR, globulin‐to‐albumin ratio; NLR, neutrophil‐to‐lymphocyte ratio; WBC, white blood cell.

During the observation period, 71 patients died, of whom 38 died due to cancer‐related disease. Univariate analyses revealed associations between OS and age (>60/≤60, year); GAR (>0.80/≤0.80); gender (male/female); GPS (2/0, 1); lymphatic invasion (presence/absence); lymph node metastasis (presence/absence); maximum tumor size (>4.0/≤4.0, cm); NLR (>2.7/≤2.7); operative bleeding (>320/≤320, mL); PNI (<45/≥45); serosal invasion (presence/absence); the serum levels of albumin (<3.5/≥3.5, g/dL), CA19‐9 (>10/≤10, U/mL), CEA (>5/≤5, ng/mL), and CRP (>1.0/≤1.0, mg/dL); TNM stage (II, III/I); and venous invasion (presence/absence) (Table 3). Multivariate analysis using the results of univariate analyses revealed that a poor OS was significantly associated with the GAR (>0.80/≤0.80) (hazard ratio [HR], 2.305; 95% CI, 1.122‐4.735; *P *=* *0.023), as well as lymph node metastasis (presence/absence) (HR, 2.417; 95% CI, 1.077‐5.426; *P *=* *0.032), neutrophil‐to‐lymphocyte ratio (>2.7/≤2.7) (HR, 2.368; 95% CI, 1.138‐4.930; *P *=* *0.002), and serosal invasion (presence/absence) (HR, 3.443; 95% CI, 1.048‐11.31; *P *=* *0.042). (Table 3).

**Table 3:** Univariate and multivariate analyses in relation to overall survival

Variable	Univariate			Multivariate		
	*P*‐value	HR	95% C.I.	*P*‐value	HR	95% C.I.
Age (>60/≤60, years)	**0.013**	2.474	1.212‐5.047	0.083	2.135	0.906‐5.034
Albumin (≤3.5/>3.5, g/dL)	**<0.001**	3.070	1.763‐5.348	0.831	1.106	0.437‐2.801
BMI (≤21.0/>21.0, kg/m^2^)	0.050	1.713	1.000‐2.934			
CA19‐9 (>10/≤10, U/mL)	**0.012**	1.961	1.163‐3.305	0.449	1.274	0.681‐2.384
CEA (>5/≤5, ng/mL)	**0.007**	2.377	1.262‐4.480	0.135	1.792	0.834‐3.850
CRP (>1.0/≤1.0, mg/dL)	**0.007**	2.898	1.344‐6.251	0.444	0.533	0.107‐2.667
GAR (>0.8/≤0.8)	**<0.001**	3.083	1.798‐5.287	**0.023**	2.305	1.122‐4.735
Gender (Male/Female)	**0.038**	1.988	1.039‐3.802	0.569	1.247	0.583‐2.665
Globulin (>3.0/≤3.0, g/dL)	0.235	1.374	0.814‐2.320			
GPS (2/0, 1)	**0.004**	3.880	1.542‐9.760	0.552	1.794	0.262‐12.29
Lymphatic invasion (presence/absence)	**0.003**	2.412	1.350‐4.312	0.375	0.639	0.238‐1.717
Lymph node metastasis (presence/absence)	**<0.001**	3.587	2.098‐6.130	**0.032**	2.417	1.077‐5.426
Maximum tumor size (4.0>/≤4.0, cm)	**0.004**	2.209	1.295–3.767	0.629	0.837	0.407‐1.721
NLR (>2.7/≤2.7)	**<0.001**	2.721	1.598‐4.634	**0.002**	2.859	1.453‐5.626
Number of tumor (≥2/1)	0.086	1.847	0.916‐3.726			
Operative bleeding (>320/≤320, mL)	**0.013**	1.950	1.154‐3.295	0.207	1.496	0.800‐2.798
Pathological differentiation (Others/well or moderately)	0.816	1.065	0.629‐1.802			
Platelet count (>20/≤20, ×10^4^/mm^3^)	0.146	0.677	0.401‐1.145			
Postoperative complication (CD grade II‐V/0, I)	0.198	1.453	0.823‐2.565			
Prognostic nutritional index (<45/≥45)	**<0.001**	2.606	1.540‐4.411	0.852	0.925	0.407‐2.100
Serosal invasion (presence/absence)	**<0.001**	4.710	2.737‐8.103	**0.042**	3.443	1.048‐11.31
TNM stage (II, III/I)	**<0.001**	4.605	2.658‐7.978	0.702	1.229	0.340‐4.965
Venous invasion (presence/absence)	**0.002**	2.390	1.391‐4.108	0.358	0.665	0.279‐1.586
WBC count (>7.0/≤7.0, ×10^3^/mm^3^)	0.452	1.242	0.707‐2.181			

The bold of *P*‐value means statistical significant value.

95% C.I., 95% confidence interval; HR, hazard ratio; BMI, body mass index; CA19‐9, carbohydrate antigen 19‐9; CD, Clavian‐Dindo; CEA, carcinoembryonic antigen; CRP, C‐reactive protein; GAR, globulin‐to‐albumin ratio; GPS, Glasgow prognostic score; NLR, neutrophil‐to‐lymphocyte ratio; TNM, Tumor‐node‐metastasis; WBC, white blood cell.

**Figure 2:**
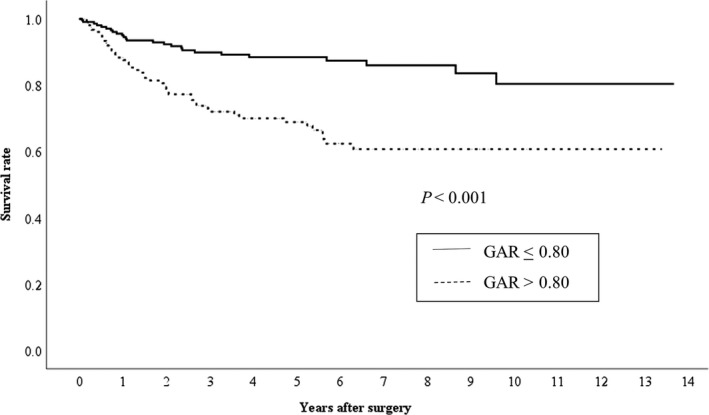
Relationship between the two globulin‐to‐albumin ratio (GAR) groups (GAR ≤ 0.80 and GAR > 0.80 from top to bottom) and overall survival in patients undergoing curative resection for gastric cancer

**Figure 3:**
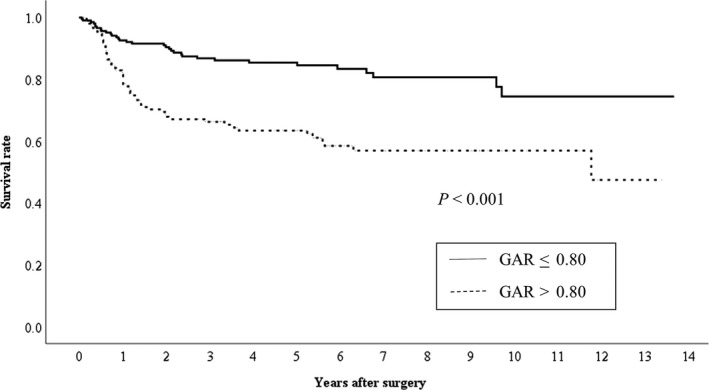
Relationship between the two globulin‐to‐albumin ratio (GAR) groups (GAR ≤ 0.80 and GAR > 0.80 from top to bottom) and relapse‐free survival in patients undergoing curative resection for gastric cancer

**Figure 4:**
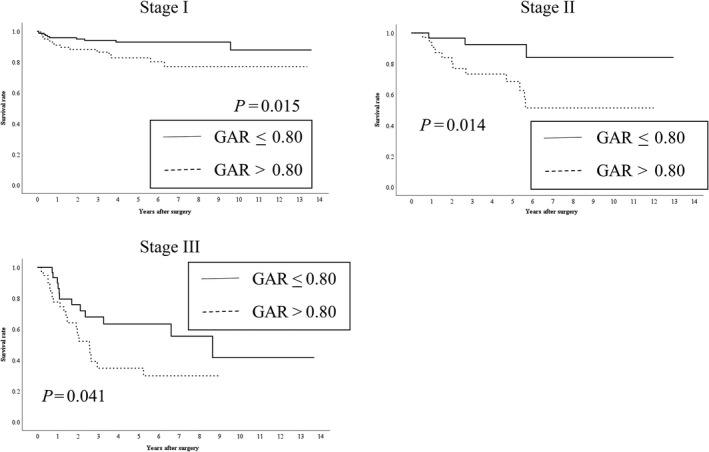
Relationship between the two globulin‐to‐albumin ratio (GAR) groups (GAR ≤ 0.80 and GAR > 0.80 from top to bottom) and overall survival in patients undergoing curative resection for each stage of gastric cancer (I/II/III)

**Figure 5:**
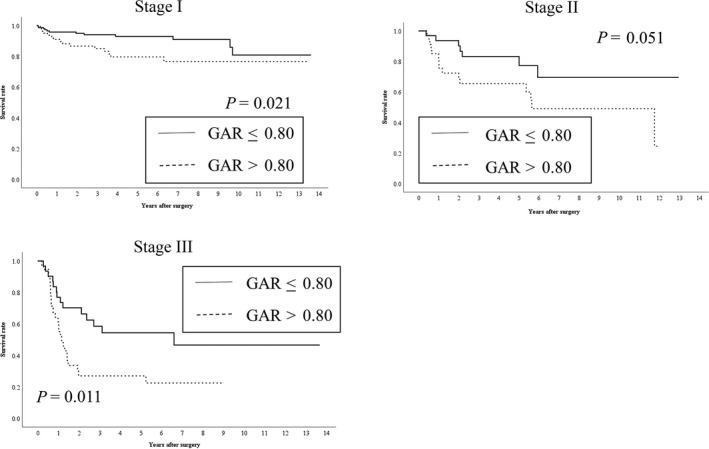
Relationship between the two globulin‐to‐albumin ratio (GAR) groups (GAR ≤ 0.80 and GAR > 0.80 from top to bottom) and relapse‐free survival in patients undergoing curative resection for each stage of gastric cancer (I/II/III)

The median and maximum survival periods were 1,425 days and 4,989 days, respectively, with a mean survival period of 1,636 ± 1,279 days. Kaplan–Meier analyses with log‐rank tests revealed a significant difference in OS according to the GAR (≤0.8 vs >0.8, *P *<* *0.001) (Figure 2). Similarly, there was a significant difference in RFS according to the GAR (≤0.8 vs >0.8, *P *<* *0.001) (Figure 3). There was a significant difference between the two groups (GAR ≤ 0.8 vs GAR > 0.8) in OS of each stage of GC patients (I/II/III) (Figure 4). With regard to RFS, although there was no significant difference between the two groups (GAR ≤ 0.8 vs GAR > 0.8) in RFS of stage II GC patients, there was a significant difference in RFS of both stage I and III GC patients (Figure 5).

## 4 DISCUSSION

Although our study included a small population of patients with an advanced TNM stage, the GAR was significantly associated with TNM stage. In addition, tumor markers and venous invasion were significantly associated with a high GAR. Previous studies have indicated that these two characteristics are associated increased angiogenesis, lymph node metastasis, and distant metastasis in GC patients.^16^, ^17^, ^18^, ^19^, ^20^ In fact, OS in the high GAR group was significantly poorer than that in the low GAR group. These facts adequately showed that the GAR can reflect not only tumor progression but also the malignant potential of GC.

Multivariate analyses revealed that the GAR was closely associated with the postoperative outcome of GC patients as well as age and TNM stage. However, the mechanism underlying the relationship between a high GAR and poor outcome of patients with a cancer is poorly understood.

It is well known that tumor necrosis factor (TNF), interleukin‐1 (IL‐1), IL‐6, and interleukin‐10 (IL‐10) are correlated with cancer‐related inflammation.^21^, ^22^ IL‐10, in particular, suppresses components of the antitumor immune system such as cytotoxic T lymphocytes and natural killer cells in cancer patients.^23^ In addition, these cytokines induce the production of various acute‐phase proteins and these proteins which are contained in the serum globulin fraction.^24^, ^25^, ^26^, ^27^, ^28^ Interestingly, it has been reported that these cytokines and acute‐phase proteins are associated with the prognosis of cancer patients.^29^, ^30^, ^31^, ^32^, ^33^, ^34^, ^35^, ^36^, ^37^ These facts suggest that an elevated serum globulin level may reflect an enhancement of tumor progression and distant metastasis due to an alteration of tumor cell biology and suppression of antitumor immunity. On the other hand, albumin plays an important role in suppressing the growth of cancer cell through its antioxidant effect and stabilization of DNA replication.^38^ Malnutrition and inflammation suppress albumin synthesis and affect the survival of cancer patients.^31^, ^39^ In fact, it has been reported that hypoalbuminemia in cancer patients is associated with poor outcome.^31^, ^38^, ^39^ In fact, patients in the high GAR group (>0.80) had decreased serum albumin level and poor outcome after curative surgery compared as low GAR group (Table 2 and Figure 2). Thus, these facts support the contention that a high GAR is strongly associated with the malignant potential of GC, and thus with outcome after curative resection.

Although our results indicated that the GAR can predict outcome after curative resection for patients with GC, it is unclear how the GAR could be applied for such patients in clinical practice. Especially, GAR significantly stratified postoperative outcome of stage II and III of GC patients (Figures 4 and 5). These results suggest that preoperative high GAR (> 0.80) would be a good indication for the new postoperative chemotherapies such as S‐1 plus oxaliplatin and capecitabine plus oxaliplatin in stage II and III of GC patients. Several previous studies have reported that S‐1 plus oxaliplatin and capecitabine plus oxaliplatin were applicable and safe as a new postoperative chemotherapy for stage II and III of GC patients.^40^, ^41^, ^42^ As a bias of this study, all stage II and III of GC patients did not receive postoperative chemotherapy using S‐1. Furthermore, the direct comparison between S‐1 and these new postoperative chemotherapies such as S‐1 plus oxaliplatin and capecitabine plus oxaliplatin in stage II and III of GC patients undergoing curative resection was not performed. However, synergy effect of combining S‐1 with other drugs would contribute to improve the outcome of stage II and III of GC patients with high GAR (>0.80). Further study regarding with these new postoperative chemotherapies should be required to improve their superiority. Therefore, considering for the patient's general status, these new chemotherapies should be considered for a good indication of postoperative chemotherapy for stage II and III of GC patients with a high GAR (>0.80).

Interestingly, GAR significantly stratified OS and RFS of stage I GC patients (Figures 4 and 5). The recurrence rate in patients with early stage GC was reported to be 1.4%‐3.0%.^43^, ^44^ Previous study reported that submucosal layer invasion, lymph node metastasis, and pathological differentiation (tub1 and tub2) were the risk factors for tumor recurrence after surgery for early stage GC.^43^ On the other hand, another previous study revealed that high age, male, lymphovascular invasion, stage IB (proper muscle invasion or lymph node metastasis), perineural invasion, and elevated tumor marker were independent poor prognostic factors for RFS.^44^ Although we did not investigate the relationship between GAR and these clinical characteristics of stage I GC which was associated with tumor recurrence, our result indicated that preoperative high GAR (>0.80) was useful for predicting poor prognosis of RFS in stage I GC patients in comparison with low GAR (<0.80). Therefore, as our new significant finding, preoperative high GAR (>0.80) is an indication of tight postoperative surveillance for stage I GC patients to improve the outcome.

The limitations of our study should be acknowledged. This was a retrospective study conducted at a single institution. The 11 patients among 376 patients undergoing surgery for GC had liver cirrhosis. Because GC patients with liver cirrhosis were small in number (11/376, 2.9%), it was considered that liver cirrhosis would affect less influence on our results. Although cytokines reflect cancer‐related inflammation, cytokine‐related characteristics were not measured in the present study. However, targeting of GC patients who had undergone curative surgery would have partly reduced the degree of bias attributable to clinical characteristics and general status. In order to resolve these problems, further studies, both prospective and using propensity score matching, will be required.

In summary, the present study has shown that the preoperative GAR can predict the outcome after curative resection for patients with GC. On the basis of these results, the GAR can be considered an indicator of appropriate adjuvant treatments for GC patients who undergo curative resection.

## DISCLOSURE

Ethical Statements: This study had the approval of the Institutional Review Board (provided ID number: 28031) and conformed to the Ethical Guidelines for Clinical Research of the Ministry of Health, Labour and Welfare, Japan (http://www.mhlw.go.jp/seisakunitsuite/bunya/hokabunya/kenkyujigyou/i-kenkyu/index.html).

Conflict of Interest: Authors declare no Conflict of Interests for this article.
